# Laparoscopic Reduction of Ileo-Ileal Intussusception in an Infant Operated for Wilms Tumor

**Published:** 2015-09-01

**Authors:** Yousuf Aziz Khan, Esmaeel Taqi, Suad Abul

**Affiliations:** Department of Pediatric Surgery, Ibn Sina Hospital of Surgical Specialties, Al-Sabah Health Region, Safat – 13115, Kuwait

**Dear Sir,**

A 55-day-old male baby underwent an exploratory laparotomy and a radical left nephro-ureterectomy with peri-hilar and para-aortic lymph nodal dissection for a left sided Wilms’ tumor. Postoperative course was smooth until 4thpostoperative day when mild abdominal distension and bilious nasogastric (NG) tube drainage of 95 ml was noticed. An x-ray abdomen was inconclusive while ultrasound (US) abdomen revealed a bowel mass with a target sign at the right pelvic region suggestive of intussusception. A trial of water-soluble contrast enema reduction was given which showed hold up of contrast at the ileum, followed by free passage of contrast into the normal caliber ileum. The intussusception mass was not visualized at subsequent US. The patient’s clinical condition did not improve with NG tube draining 160 ml bilious aspirate following 24 hours and a repeat US showed recurrence of intussusception. It was then decided for laparoscopic exploration. An ileo-ileal intussusception was found which was easily reduced laparoscopically and bowel appeared viable (Fig.1). The postoperative course was uneventful then. The histopathology reported COG Stage I Wilms’ tumor (triphasic) with favorable histology.

**Figure:**
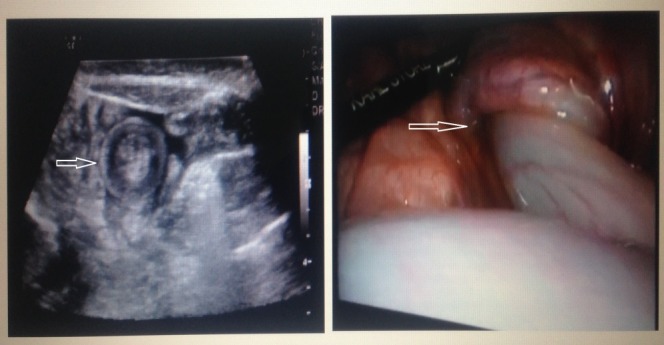
Figure 1: Showing ‘target’ sign of intussusception on US and peroperative picture of laparoscopic reduction of intussusception.

Surgical procedures involving extensive retroperitoneal dissection and bowel manipulation cause impairment of bowel innervation and peristalsis, as in resection of Wilms’ tumor, neuroblastoma, pull-through for Hirschsprung’s disease, Ladd’s procedure, diaphragmatic hernia repair and fundoplication are reported to have a high incidence of postoperative intussusception (POI).[1-3]

The classical triad of intussusception is usually not seen and in the presence of expectant postoperative ileus, the diagnosis can be easily overlooked which can increase morbidity and mortality.[3] Bilious vomiting, prolonged and excessive NG bilious aspirate and abdominal distension are usually the common findings reported.[2,4] A similar presentation was seen in our case. When a smooth postoperative course is interrupted by deterioration in clinical symptoms and signs, POI should be suspected. In a case of atypical postoperative ileus, radiologic investigations as US should not be delayed; it is highly accurate, supportive diagnostic tool in cases of POI as in primary intussusception.[2,4]

Reduction of intussusception is the mainstay of treatment. In failed non-operative reduction of intussusception, laparoscopy is a safe and effective operative.(5) In the index case, same approach was used. In case of recurrence, re-laparoscopy can be performed for reduction of intussusception rather than laparotomy.

## Footnotes

**Source of Support:** Nil

**Conflict of Interest:** None declared

